# Prevalence and risk factors for impaired kidney function in the district of Anuradhapura, Sri Lanka: a cross-sectional population-representative survey in those at risk of chronic kidney disease of unknown aetiology

**DOI:** 10.1186/s12889-019-7117-2

**Published:** 2019-06-14

**Authors:** Thilanga Ruwanpathirana, Sameera Senanayake, Nalika Gunawardana, Asoka Munasinghe, Samitha Ginige, Deepa Gamage, Jagath Amarasekara, Buddi Lokuketagoda, Pubudu Chulasiri, Sarath Amunugama, Paba Palihawadana, Ben Caplin, Neil Pearce

**Affiliations:** 1 Epidemiology Unit, Ministry of Health, 231, De Saram Place, Colombo, 10 Sri Lanka; 2 World Health Organization, Country Office, Colombo, Sri Lanka; 3 Regional Director of Health Office, Anuradhapura, Sri Lanka; 4 Ministry of Health, Colombo, Sri Lanka; 5 Department of Renal Medicine, University College London, London, UK; 6 Department of Medical Statistics, Faculty of Epidemiology and Population Health, London School of Hygiene and Tropical Medicine, London, UK

**Keywords:** Sri Lankan agricultural nephropathy, Chronic interstitial nephritis in agricultural communities, Chronic kidney disease of unknown cause

## Abstract

**Background:**

Over the last 20 years there have been reports of a form of chronic kidney disease of unknown cause (CKDu) affecting rural communities in the North Central Province of Sri Lanka. Valid prevalence estimates, using a standardised methodology, are needed to assess the burden of disease, assess secular trends, and perform international comparisons.

**Methods:**

We conducted a cross-sectional representative population survey in five study areas with different expected prevalences of CKDu. We used a proxy definition of CKDu involving a single measure of impaired kidney function (eGFR< 60 mL/min/1.7m^2^, using the CKD-Epi formula) in the absence of hypertension, diabetes or heavy proteinuria.

**Results:**

A total of 4803 participants (88.7%) took part in the study and 202 (6.0%; 95% CI 5.2–6.8) had a low eGFR in the absence of hypertension, diabetes and heavy proteinuria and hence met the criteria for proxy CKDu. The proportion of males (11.2%; 95% CI 9.2–13.1) were triple than the females (3.7%; 95% CI 2.9–4.5). Advancing age and history of CKD among parents or siblings were risk factors for low GFR among both males and females while smoking was found to be a risk factor among males.

**Conclusions:**

These data, collected using a standardised methodology demonstrate a high prevalence of impaired kidney function, not due to known causes of kidney disease, in the selected study areas of the Anuradhapura district of Sri Lanka. The aetiology of CKDu in Sri Lanka remains unclear and there is a need for longitudinal studies to describe the natural history and to better characterise risk factors for the decline in kidney function.

**Electronic supplementary material:**

The online version of this article (10.1186/s12889-019-7117-2) contains supplementary material, which is available to authorized users.

## Background

Chronic kidney disease of unknown aetiology (CKDu) is of major public health importance in Sri Lanka, affecting predominantly farming communities. The problem was first identified in the early 1990s among rice farmers of the North Central province [1–3], specifically in the district of Anuradhapura. The emergence of CKDu in Sri Lanka coincided with the recognition of similar kidney disease of unknown cause in agricultural communities in Central America [4]. This was seen mainly among agricultural workers (particularly sugar cane plantation workers) in the west coast of the Mesoamerican isthmus. Nicaragua and El-Salvador are the two countries which are most affected. More recently, similar reports of CKDu have emerged from the Uddanam District of Andhra Pradesh in South India [5].

Local and international researchers have proposed several hypotheses as to the causes of CKDu. Environmental pollution due to heavy metals such as cadmium [6] and arsenic [7] and agrochemicals such as glyphosate [8], excess fluorides coupled with hardness in water [9], infections such as leptospirosis and hantavirus [10] and genetic factors [3], exposure to fungal and bacterial toxins [11] and heat stress [12] are among the hypotheses. Support for these hypotheses has been mostly derived from cross-sectional studies, which have often focused on single factors, and which have not taken into account the confounding effects of other potential risk factors, or interactions between risk factors.

In Sri Lanka, CKDu has been reported to be common and increasing in prevalence [13], although robust standardized data to support these claims are lacking. Routine data sources are inadequate for accurate estimation of the prevalence and trends of CKD or CKDu. For example, routine morbidity and mortality surveillance through hospital statistics and vital registration systems do not include a specific category for CKDu, and estimates of deaths due to CKD are likely to be subjected to substantial ascertainment bias. Furthermore, community-based screening programmes in areas designated as ‘high risk’ have suffered from low or differential response rates and/or have not collected kidney function data on the entire study population (e.g. in some studies, eGFR has only been measured in those with elevated urine protein tests).

Given the public health importance of the issue of CKDu for global health, and to promote generation of comparable evidence across studies in various countries with uniform measures that ensure quality of data, an international group has been formed to address these issues, the Disadvantaged Populations eGFR Epidemiology (DEGREE) collaboration. This group has developed a protocol for cross-sectional surveys of eGFR distribution in low- and middle- income communities to provide a basis for internationally comparable estimates of the burden of CKDu [14]. The present survey was undertaken using the DEGREE protocol, to estimate the prevalence and geographical distribution of low eGFR in the district of Anuradhapura which is the district which records the highest numbers of patients with CKDu in Sri Lanka. The study also aimed to identify risk factors associated with low eGFR, with a view to investigating these further using a prospective cohort design.

## Methods

The study followed the core protocol of the Disadvantaged Populations eGFR Epidemiology Study (DEGREE) [14], but added additional questions on risk factors relevant to the Sri Lankan situation.

### Study design and study setting

**Fig. 1:**
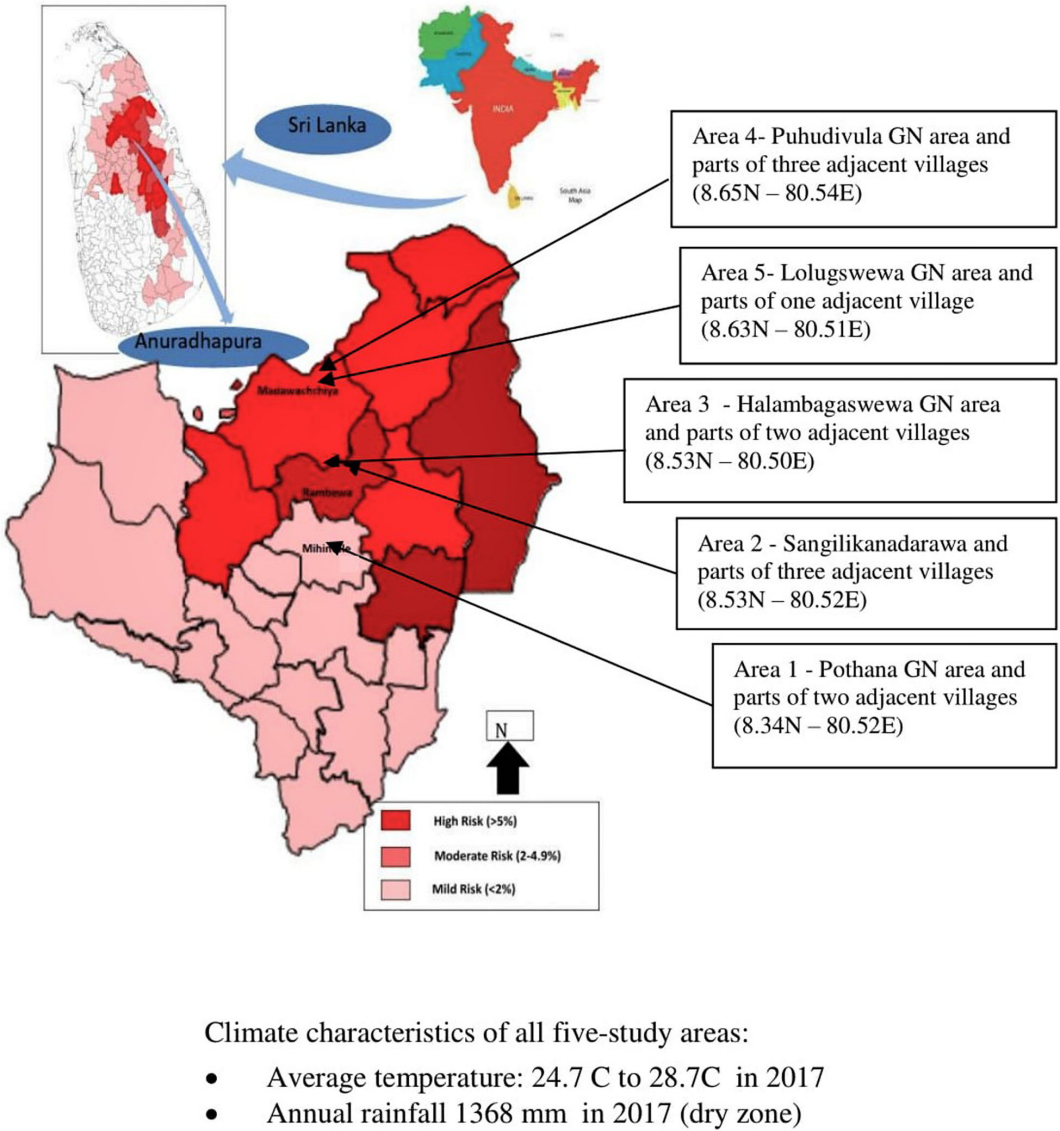
Study settings and their characteristics

**Fig. 2:**
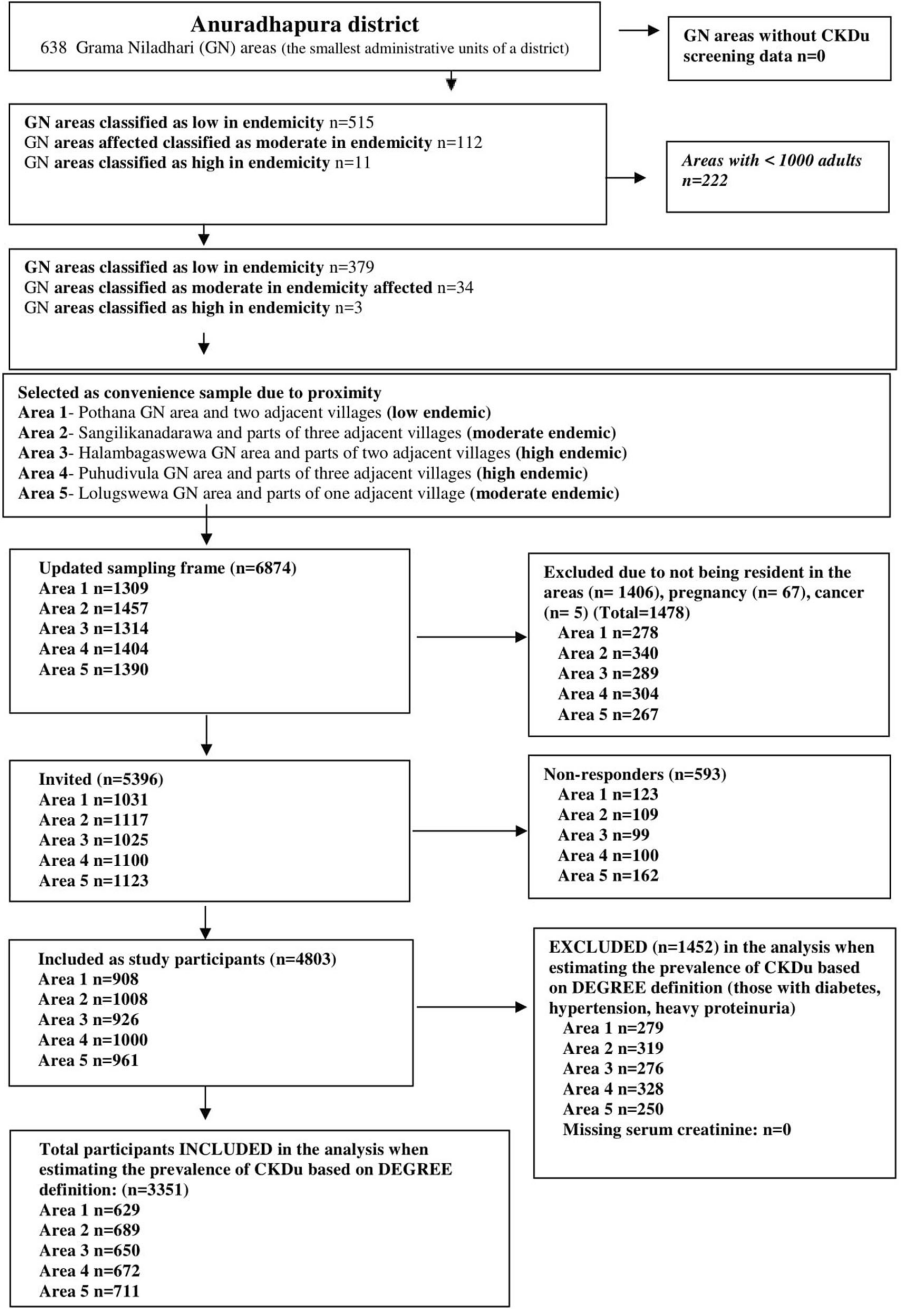
Flow diagram to select the study setting

This was a community-based cross-sectional household survey. The study was conducted in five geographically demarcated communities in the district of Anuradhapura from March to May 2017. The government routinely conducts community-based screening in Anuradhapura, and classifies the Grama Niladhari areas (the smallest administrative units of a district) into three levels of endemicity for CKD/CKDu. The five areas were selected based on the existing classification of Grama Niladhari areas as high/moderate/low levels of endemicity of CKDu with one community from the group classified as ‘low and two areas each from the groups of moderate and high (Fig. 1 and Fig. 2).

### Study participants

All adults above the age of 18 years, whose main place of residence (defined as living in the setting for at least for 5 days of the week for the past 6 months) was in the study area, were invited to take part. Exclusion criteria were: (i) pregnancy; and (ii) patients undergoing treatment for cancer. About 1000 eligible study participants were selected from each of the study areas to allow accurate estimates of the prevalence of CKDu each study area. These numbers also provide sufficient statistical power for comparisons between study areas or between population subgroups [14]. The updated voter’s lists of the selected five study areas was used in the stage of planning of the study to define the extent of the geographical area that would be needed to recruit 1000 eligible study units. All of the households in the defined study area were visited and all eligible adults were invited to take part. Study information was provided, and those giving informed written consent were recruited to the study. A team of 10 graduates from a University located in the district were trained to recruit the study participants.

### Data collection

Upon recruitment, all participants were issued a unique identifying number. Information on socio-demographic characteristics, medical history, specifically past medical history of diabetes mellitus and hypertension and other potential risk factors were collected in the home using an interviewer-administered questionnaire developed for this study, by the trained interviewers (Additional file 1). The medical and treatment records were also photographed, and were verified later by a medical doctor. Upon completion of the questionnaire, study participants were provided with a container and an instruction sheet on collecting the early morning urine sample and were requested to visit the ‘clinic’ on the following day before work for the anthropometry measurements and biological sample collection. Revisits to the houses were done to recruit any eligible study participants who were not available in the house at the time of the first visit. The ‘clinics’ were set up within the study areas in locations that were acceptable and accessible to all the villagers and about 100 were invited to each clinic. A total of 12 ‘clinics’ were conducted with 2 or 3 in each study area. A retired Public Health Inspector was recruited to coordinate the clinic data collection. In the ‘clinics’, the early morning sample of urine brought by the study participants was stored for later analysis. A team of undergraduates of the Rajarata University conducted the anthropometry measurements and samples of 3 ml of blood drawn. Measurements of blood pressure (three times 5 min apart using electronic blood pressure apparatus, sitting position), capillary random plasma glucose using a glucometer, height using stadiometer, bio-impedance outputs of body fat %, body mass index (BMI) and total body water % using a TANITA SC-240MA body composition analyzer were then conducted. The blood samples were centrifuged on-site using a portable centrifuge before all bio samples were stored in an igloo which was maintained at a temperature of 2–8 °C. The ‘clinic’ coordinator ensured proper storage and transport of biological samples to the chemical pathology laboratory of the Anuradhapura Teaching Hospital after each clinic session. In the laboratory, samples of serum (total of 2 ml stored as a single aliquot) and urine (2 ml in one aliquot) were separated for bio-banking purposes and frozen (− 20 °C).

Institutional ethics committee approval was obtained from the Ethics Review Committee of the Faculty of Medicine, University of Colombo (EC-17-031). All reports of investigations of the biological samples and antrhopometric measurements were shared with the study participants. All those newly identified with low eGFR, hypertension and diabetes were referred to the nearest hospital for treatment.

### Analysis of biological samples

Serum creatinine and urine protein: Creatinine ratios were tested in the laboratory of the Anuradhapura Teaching Hospital in a single batch. Serum creatinine was measured using assays calibrated utilizing quality controls traceable to isotope dilution mass spectrometry (IDMS) standards.

### Data analysis

A data entry form was created in the Epidata package with relevant range and consistency checks. Four data entry operators entered the data of the questionnaire, body measurements and laboratory reports identified by the unique identifying number. A medical doctor was trained to interpret photographs of medical and treatment histories and enter the verified data into the relevant data sheets. Double entry of data was conducted for data on serum creatinine and albuminuria of all study participants. The investigator conducted double entry of randomly selected clinic data and information obtained through the questionnaires to assess and to ensure the quality of data entry.

### Statistical analysis

We used the DEGREE proxy definition of CKDu based on reduced kidney function. That is:

One time measurement of eGFR < 60 mL/min/1.7m^2^ (eGFR< 60) using calculated using CKD EPI equation AND without

i. hypertension

- blood pressure > 140/90 at the time of the survey
- being on anti-hypertension drugs (any number)
- self-reported of hypertension with evidence of medical records

ii. diabetes mellitus

- Self –report of diabetes
- being on drug treatment for diabetes
- capillary random plasma glucose > 200 mg/dL at the time of survey

iii. heavy proteinuria

- albumin/creatinine ratio (ACR) ≥300 mg/g

As the DEGREE study protocol recognizes [14], this definition does not directly correspond to CKDu, because it uses a one-time measurement of eGFR. However, it forms a suitable outcome measure for community-based surveys, and should reflect the underlying prevalence of CKDu (assuming that few ‘cases’ would be reclassified on the basis of a second eGFR measurement, and that the proportion who would be reclassified would not differ markedly by study area).

Prevalences of eGFR 60–90 mL/min/1.7m^2^ (eGFR60–90), 30–60 mL/min/1.7m^2^ (eGFR30–60) and < 30 mL/min/1.7m^2^ (eGFR< 30) were then estimated by sex, age-group and study area. The prevalence of the participants with eGFR in the categories above but without hypertension, diabetes or heavy proteinuria was also estimated by the same variables. Age-standardisation to the World Health Organization World population (http://www.who.int/healthinfo/paper31.pdf) [15] was conducted to enable us to compare between study areas.

Thereafter, we examined the associations of eGFR (as a continuous variable) and eGFR< 60 by socio-demographic, life style and biological characteristics in the population restricted to those without diabetes, hypertension or heavy proteinuria. We conducted age- and sex- adjusted analyses to assess the associations between this proxy measure of CKDu and socio-demographic, life style and biological characteristics. Finally, adjusted multiple regression analyses were conducted to identify factors independently associated with eGFR< 60. The standard errors of the key effect estimates in the ‘full’ model and the corresponding standard errors in the initial models (only adjusted for age and sex) were compared to check for multicollinearity [14]. No important collinearity was observed, so the full model was used for the final analyses. Finally we conducted a further sensitivity analysis to examine the association between farming and eGFR (in the absence of diabetes, hypertension or heavy proteinuria) using a stepwise multiple regression approach.

## Results

### Characteristics of the study participants

The study included 4803 participants, with an overall response rate of 88.7%. The response rate among the eligible females (90.4%) was higher than the males (85.4%). The socio-demographic characteristics of all the study participants in each of the study areas are shown in Table 1. Nearly half of the study participants were less than 44 years old and a majority were females (68.2%). Most males (85.7%) had been occupied in farming with equal proportions reporting full time (42.6%) and part-time (43.1%) engagement. Among females, the proportion ever occupied in full-time farming was similar to males (48.5%) while those who reported part-time farming were lower (21.9%). Nevertheless, a majority (75.3%) or the whole population had been occupied in farming at some time.

**Table 1:** Socio-demographic characteristics of the study population by study area

Socio-demographic characteristics	Area 1 (*n* = 908)	Area 2 (*n* = 1008)	Area 3 (*n* = 926)	Area 4 (*n* = 1000)	Area 5 (*n* = 961)	Total (*n* = 4803)
	n (%)	n (%)	n (%)	n (%)	n (%)	n (%)
Age-group (years) (*n* = 4803)						
< 25	63 (6.9)	82 (8.1)	69 (7.5)	68 (6.8)	58 (6.0)	340 (7.1)
25–34	144 (15.9)	167 (16.6)	153 (16.5)	173 (17.3)	154 (16.0)	791 (16.5)
35–44	214 (23.6)	265 (26.3)	243 (26.2)	235 (23.5)	279 (29.0)	1236 (25.7)
45–54	161 (17.7)	216 (21.4)	188 (20.3)	212 (21.2)	200 (20.8)	977 (20.3)
55–64	181 (19.9)	165 (16.4)	161 (17.4)	180 (18.0)	166 (17.3)	853 (17.8)
65–70	92 (10.1)	75 (7.4)	63 (6.8)	75 (7.5)	70 (7.3)	375 (7.8)
> 70	53 (5.8)	38 (3.8)	49 (5.3)	57 (5.7)	34 (3.5)	231 (4.8)
Sex (*n* = 4803)						
Male	290 (31.9)	340 (33.7)	313 (33.8)	300 (30.0)	286 (29.8)	1529 (31.8)
Female	618 (68.1)	668 (66.3)	613 (66.2)	700 (70.0)	675 (70.2)	3274 (68.2)
Years of education in schools and in higher education institutes (*n* = 4803)						
No schooling	70 (7.7)	40 (4.0)	62 (6.7)	34 (3.4)	32 (3.3)	238 (5.0)
< 10	302 (33.3)	336 (33.3)	310 (33.5)	361 (36.1)	341 (35.5)	1650 (34.4)
≥ 10	536 (59.0)	632 (62.7)	554 (59.8)	605 (60.5)	588 (61.2)	2915 (60.7)
Ever occupied in farming (*n* = 4803)						
Full time farming	444 (48.9)	391 (38.8)	435 (47.0)	482 (48.2)	486 (50.6)	2238 (46.6)
Male	131 (45.2)	124 (36.5)	149 (47.6)	125 (41.7)	122 (42.7)	651 (42.6)
Female	313 (50.6)	267 (40.0)	286 (46.7)	357 (51.0)	364 (53.9)	1587 (48.5)
Part time farming	299 (32.9)	295 (29.3)	255 (27.5)	280 (28.0)	248 (25.8)	1377 (28.7)
Male	123 (42.4)	147 (43.2)	122 (39.0)	140 (46.7)	127 (44.4)	659 (43.1)
Female	176 (28.5)	148 (22.2)	133 (21.7)	140 (20.0)	121 (17.9)	718 (21.9)
No	165 (18.2)	322 (31.9)	236 (25.5)	238 (23.8)	227 (23.6)	1188 (24.7)
Male	36 (12.4)	69 (20.3)	42 (13.4)	35 (11.7)	37 (12.9)	219 (14.3)
Female	129 (20.9)	253 (37.9)	194 (31.6)	203 (29.0)	190 (28.1)	969 (29.6)

### Prevalence of impaired kidney function in the absence of known causes of CKD

A total of 1262 (26.3, 95% CI 25.0–27.5) of the study population had hypertension, while 470 had diabetes mellitus (9.7, 95% CI 8.9–10.6). The number with both hypertension and diabetes mellitus was 280. A total of 110 who were found to be suffering from heavy proteinuria, also suffered from diabetes or hypertension Thus, when the analysis was restricted to those without hypertension, diabetes and heavy proteinuria, a total of 1452 people were excluded from the analyses, including 177 ‘cases’ of eGFR< 60 mL. (Additional file 2).

Overall, 202 of 3351 (6.0%; 95% CI 5.2–6.8) study participants had a low eGFR in the absence of hypertension, diabetes and heavy proteinuria and hence met the criteria for proxy CKDu. The prevalence in males (11.2%; 95% CI 9.2–13.1) was triple that the females (3.7%; 95% CI 2.9–4.5). (Table 2.) Areas 2, 3, 4 and 5 had higher crude rates of eGFR< 60 without hypertension, diabetes or heavy proteinuria compared to Area 1. This pattern more or less conformed to what was expected based on prior information which was used to select the study areas, in which Area 1 was expected to be as low endemicity. However, we also expected that Areas 2 and 5 would be moderate endemicity and 3 and 4 would be high endemicity, and these expectations were not clearly verified in the study results. Among males, age-standardized rates of eGFR< 60 without hypertension, diabetes or heavy proteinuria was higher in areas 3 and 4 while among females, the corresponding rate was higher in Area 3. (Additional file 3). Prevalence of eGFR< 60 according to occupation and lifestyle factors in the absence of hypertension, diabetes and proteinuria by sex is indicated in the Additional file 4.

**Table 2:** Prevalence of eGFR< 60 in the absence of hypertension, diabetes and proteinuria by age, sex and study area

Prevalence	Area 1 (*n* = 629)		Area 2 (*n* = 689)		Area 3 (*n* = 650)		Area 4 (*n* = 672)		Area 5 (*n* = 711)		Total (*n* = 3551)	
	%	Cl	%	Cl	%	Cl	%	Cl	%	Cl	%	Cl
eGFR < 60 (*n* = 202)												
18–30 years (*n* = 728)	0		0.7	0.0–2.1	0.7	0.0–2.1	0		0		0.3	0.0–0.7
31–40 years (*n* = 955)	0		2.8	0.3–5.3	0.6	0.0–1.8	0.5	0.0–1.6	0.4	0.0–1.3	0.8	0.2–1.4
41–50 years (*n* = 788)	1.6	0.0–3.9	4.1	1.3–6.9	4.0	1.1–7.0	5.4	1.9–8.8	4.2	1.1–7.3	4.0	2.6–5.3
51–60 years (*n* = 502)	3.5	0.1–6.9	6.4	1.7–11.2	9.1	3.3–14.8	8.3	2.7–13.9	12.1	6.0–18.1	7.8	5.6–10.1
61–70 years (*n* = 297)	13.2	4.9–21.5	21.4	10.3–32.5	22.4	11.3–33.4	43.1	29.9–56.2	25.9	15.9–36.0	24.9	20.1–29.7
> 70 (*n* = 81)	30.7	11.7–49.7	40.0	11.9–68.1	28.5	1.5–55.6	58.3	37.0–79.6	37.5	10.8–64.1	40.0	29.9–50.0
All (*n* = 3351)	3.7	2.2–5.1	5.6	3.9–7.4	5.4	3.6–7.1	8.5	6.4–10.6	6.7	4.9–8.6	6.0	5.2–6.8
eGFR < 60 among males (*n* = 116)												
18–30 years (*n* = 162)	0	0.0	0	0.0	0	0.0	0	0.0	0	0.0	0	0.0
31–40 years (*n* = 246)	0	0.0	3.4	0.0–8.1	2.3	0.0–7.0	0	0.0	1.8	0.0–5.5	1.6	0.0–3.2
41–50 years (*n* = 288)	2.9	0.0–8.9	2.8	0.0–6.7	9.3	2.6–16.1	8.9	1.2–16.6	3.8	0.0–9.2	5.9	3.2–8.6
51–60 years (*n* = 179)	8.1	0.0–17.3	20.0	6.0–33.9	13.5	1.9–25.1	29.4	13.3–45.5	25.0	10.1–39.8	19.0	13.2–24.8
61–70 years (*n* = 125)	11.7	0.3–23.2	36.8	12.9–60.7	41.7	20.4–62.9	50.0	28.4–71.6	50.0	28.4–71.6	36.0	27.5–44.5
> 70 (*n* = 37)	42.8	0.0–92.3	14.3	0.0–49.2	50.0	0.0–100.0	60.0	23.1–96.9	42.8	0.0–92.2	43.2	26.5–59.9
All (*n* = 1037)	5.8	2.4–9.1	8.0	4.5–11.5	11.8	7.5–16.2	17.5	12.0–23.0	13.2	8.5–17.9	11.2	9.2–13.1
eGFR < 60 among females (*n* = 86)												
18–30 years (*n* = 566)	0	0.0	0.9	0.0–2.6	0.8	0.0–2.5	0	0.0	0	0.0	0.3	0.0–0.8
31–40 years (*n* = 709)	0	0.0	2.4	0.0–5.0	0	0.0	0.6	0.0–1.9	0.6	0.0–1.7	0.7	0.1–1.3
41–50 years (*n* = 500)	2.5	0.0–6.0	5.5	1.2–9.9	1.0	0.0–3.1	3.7	0.1–7.5	3.6	0.1–7.2	3.4	1.8–4.9
51–60 years (*n* = 323)	0	0.0	2.9	0.0–7.2	7.3	0.2–14.3	3.6	0.0–8.6	10.7	3.5–17.8	4.9	2.5–7.3
61–70 years (*n* = 172)	13.5	1.9–25.1	12.9	0.4–24.4	6.9	0.0–16.7	37.9	19.1–56.7	13.0	2.9–23.2	16.3	10.7–21.8
> 70 (*n* = 44)	41.7	8.9–74.4	66.7	12.5–100.0	12.5	0.0–42.0	50.0	16.8–83.2	33.3	0.0–87.5	40.9	25.8–56.0
All (*n* = 2314)	2.7	1.2–4.2	4.4	2.5–6.3	2.1	0.7–3.4	4.9	3.0–6.9	4.1	2.4–5.8	3.7	2.9–4.5

Multiple logistic regression was used to identify independent associations between socio-demographic, life style and biological characteristics and eGFR< 60 (Table 3) among the population without diabetes, hypertension and heavy proteinuria. Advanced age, history of CKD among parents or siblings and living in areas 2 and 4 were significant risk factors for eGFR< 60 in fully adjusted models in both sexes.

**Table 3:** Multiple logistic regression analysis of sociodemographic, life style and biological characteristics associated with eGFR< 60 in the absence of hypertension, diabetes and proteinuria

Variable	Males					Females				
	N	Adjusted Odds Ratio	95% CI		*p* value	N	Adjusted Odds Ratio	95% CI		*p* value
Age	1037	1.1	1.08	1.14	< 0.001	2314	1.1	1.1	1.1	< 0.001
Number of years of education in schools and in higher education institutes	1037	0.9	0.9	1.0	0.07	2314	1.0	0.9	1.0	0.16
Study area										
Area 1	190	1.0				439	1.0			
Area 2	236	2.6	1.0	6.7	0.04	453	2.8	1.2	6.8	0.02
Area 3	219	2.5	1.0	6.1	0.05	431	1.1	0.4	2.9	0.92
Area 4	188	3.9	1.6	9.5	0.003	484	3.5	1.4	8.5	0.006
Area 5	204	3.5	1.4	8.8	0.007	507	2.2	0.9	5.3	0.08
History of CKD among parents or siblings										
Yes	345	2.4	1.4	4.1	0.001	725	1.8	1.1	3.0	0.02
No	690	1.0				1589	.01			
Occupation										
Ever occupied in any farming and duration										
No farming	179	1.0				829	1.0			
Part time farming for < 10 yrs	145	1.4	0.3	6.8	0.65	157	1.0	0.3	4.0	0.97
Part time farming for ≥10 yrs	296	2.0	0.6	7.0	0.28	253	1.4	0.6	3.3	0.40
Full time farming for < 10 yrs	57	0.4	0.0	2.1	0.30	291	0.3	0.0	2.1	0.20
Full time farming for ≥10 yrs	360	2.1	0.6	7.8	0.25	784	1.5	0.7	3.3	0.34
Use of fertilizers/ Weedicides/ Pesticides										
Yes	739	0.9	0.4	1.9	0.85	699	0.6	0.3	1.0	0.05
No	298	1.0				1615	1.0			
Life style factors										
Ever smoking										
Yes	523	1.9	1.1	3.2	0.02	16	3.1	0.5	19.7	0.24
No	514	1.0				2298	1.0			
Alcohol ever use										
Yes	744	0.8	0.4	1.4	0.38	43	1.5	0.3	6.8	0.57
No	293	1.0				2271	1.0			
Deep-wells as the drinking water source										
Yes	793	0.5	0.2	1.4	0.20	1721	0.5	0.2	1.3	0.18
No	244	1.0				593	1.0			
Shallow wells as the drinking water source										
Yes	84	0.4	0.1	1.3	0.12	169	0.5	0.2	1.9	0.34
No	953	1.0				2145	1.0			
Tube well as the drinking water source										
Yes	104	0.8	0.3	2.1	0.70	239	0.8	0.3	2.1	0.70
No	933	1.0				2075	1.0			
Amount of water consumed per day										
Less than 3 l	407	1.0			0.06	1524	1.0			0.28
3 or more liters	630	1.7	1.0	2.8		790	1.3	0.8	2.3	
Work outside exposed to the sun										
Less than 20 h per week	458	1.0			0.29	1698	1.0			0.87
20 h or more per week	579	0.8	0.4	1.3		616	1.0	0.6	1.9	
Snake bite (any snake)										
Yes	82	1.7	0.8	3.4	0.17	92	1.8	0.7	4.7	0.22
No	955	1.0				2222	1.0			
Biological factors										
Body Mass Index (kg/m^2^)	1037	1.0	0.9	1.1	0.78	2314	1.1	1.0	1.2	0.11
Body fat %	1037	1.0	1.0	1.1	0.35	2314	1.0	0.9	1.0	0.22
Body water %	1037	1.1	1.0	1.2	0.09	2314	1.0	0.9	1.1	0.58

We assessed residual confounding in the association between eGFR (among those with no diabetes, or hypertension or heavy protienuria) and ever occupied in farming by controlling for potential confounders; we found that eGFR greatly reduces when controlled for sex and age with the reduction when controlled only for age being higher than the reduction when controlled only for sex. However, there was little change in the findings when they were controlled for the other variables considered (Additional file 5).

## Discussion

We conducted a population-representative cross-sectional survey of eGFR in area at risk of CKDu, and observed that 12% of the study population had eGFR< 60. When restricted to those without known risk factors or clinical evidence of known forms of CKD, a proxy definition for CKDu, this prevalence estimate fell to 6%. It is also worth noting that clinically important renal impairment commonly occurs in middle-age, but that it is rare for it to occur in the absence of known risk factors for CKD.

Lack of a uniform case definition used for CKDu in previous studies in Sri Lanka hinders direct comparison of results with previous estimates of prevelance. However, according to a large community-based study conducted in the endemic areas in 2010 among 4777 adults, the prevalence of CKDu in three endemic districts of Anuradhapura, Polonnaruwa and Badulla were 15, 20.6 and 22.9%, respectively based on a case definition the presence of an ACR ≥ 30 mg/g on two occasions in combination with exclusion of other causes of CKD [2]. Furthermore, Athuraliya et al. reported an overall prevalence of CKDu of 4.2% among 2600 adults in Medawachchiya in Anuradhapura district in 2011 based on a similar case definition [16].

Finally a cross-sectional survey of 886 individuals in Anuradhapura district reported a proxy indicator of CKDu at an overall prevalence of 7.3% [17]. Only in the first of these studies was kidney function also tested (in those with proteinuria) and less than half of those who were tested had an eGFR< 60. However proteinuria is not typical of CKDu in other parts of the world [17, 18], and 62% of the participants with eGFR< 60 in our study had an ACR < 30 mg/g, and would have been misclassified if primary screening had been based on the presence of proteinuria when estimating the population prevalence of impaired kidney function.

Although direct comparisons internationally are difficult (due to differing study designs and population age structures) the prevalence estimates of the current study is probably at least 5 times what would be expected in high-income countries. For example, US population-based estimates of prevalence of an eGFR< 60 mL/min in those without hypertension or diabetes are ~ 1% in those under 60-years of age [19]. However these Sri Lankan estimates compare favourably with estimates from Central America, where overall rates of eGFR< 60 in those under 60 years of age have been reported to be as high as 18% in some communities (where hypertension and diabetes are relatively uncommon) [18]. Consistent with a less aggressive form of CKDu in South Asia compared with Mesoamerica, is the age of those affected, with the proxy CKDu outcome being rare in those under the age of 45 in this study.

Therefore, although the previous studies above have provided evidence that a problem of CKDu exists in Sri Lanka, the findings are not comparable across studies, or with findings from other countries. The findings of the current study therefore represent the first data from Sri Lanka with a population collected using internationally standardized protocols, and a standardised proxy definition of CKDu.

Risk factors for impaired kidney function in this study include well-known risk factors for low eGFR across all populations such as age and smoking. Consistent with CKDu in other parts of the world, male sex was associated with eGFR< 60 in the absence of risk factors or clinical evidence of known forms of CKD [18]. However, no associations were identified with outdoor/agricultural work, agrochemical exposure or water source. Taken together, these findings do not provide clear insight as to the cause(s) and furthermore do not provide clear evidence that the CKDu in the South Asia and Mesoamerica represent the same disease entity.

This study has several other strengths including high response rates, batched and internationally referenced laboratory measures and moderately large sample size. There are also a number of weaknesses that should be acknowledged.

It should be noted that CKDu has been identified to be endemic only in selected regions in the country and that this study was conducted in one of them. Thus, the findings of the study cannot be generalized to other parts of the country. Despite the high response rates in both sexes, the proportion of males among the study participants was less than 50%. Enlistment in the Armed Forces is common amongst men in Anuradhapura district. Although these men still consider the study communities to be their home, they did not meet the residence criteria in the protocol and hence were excluded from the sampling frame. Similarly, adult males in the district are commonly migrating to urban areas to take advantage of improved employment opportunities. However, we also estimated age-standardised rates in order to obtain valid prevalence comparisons between males and females, and between different areas.

Using a single measure of eGFR to define cases will lead to the inclusion of participants suffering acute kidney injury (AKI) among the cases, using our proxy definition of CKDu. This misclassification bias is likely to be rare, but may nonetheless inflate our prevalence estimates to some extent.

Conversely we may have underestimated the prevalence of eGFR< 60 as the CKD-Epi equation is not validated in the Sri Lankan population. By analogy with other South Asian populations this equation is likely to have overestimated the actual GFR. This means our prevalence estimates of CKDu are actually likely to be underestimates, although it is unlikely that the extent of underestimation would differ by area or by population subgroups.

Furthermore, the small sample size in some cells in Table 3 could be the reason that known risk factors did not reach statistical significance in logistic regression (e.g. smoking in females).

## Conclusions

In conclusion, we conducted a cross-sectional population-representative study and identified a high prevalence of impaired kidney function with no obvious cause in the selected study areas in Anuradhapura, Sri Lanka. These data collected using a standardised methodology can be used for comparisons with other regions and countries as well as document secular trends. The aetiology of CKDu in Sri Lanka remains unclear and there is a need for longitudinal studies to describe the natural history and better characterise risk factors for this disease.

## Additional files

Additional file 1: Questionnaire. Questionnaire used to collate information necessary to development the research. (PDF 195 kb)

Additional file 2: Exclusion criteria. Table detailing the prevalence of the exclusion criteria by sex and study area (DOCX 29 kb)

Additional file 3: Age-standardized prevalence rates of CKDu. Table detailing the prevalence of low eGFR in the absence of hypertension, diabetes and proteinuria by sex, age and the study (DOCX 34 kb)

Additional file 4: CKDu prevalence according to occupation and lifestyle factors. Table detailing the prevalence of eGFR< 60 according to occupation and lifestyle factors in the absence of hypertension, diabetes and proteinuria by sex (DOCX 28 kb)

Additional file 5: Sensitivity analysis. Table detailing the results of the sensitivity analysis on the association of eGFR (in the absence of diabetes, or hypertension or heavy protienuria) and ever occupied in of farming (DOCX 35 kb)

## Data Availability

The datasets used and/or analysed during the current study available from the corresponding author on reasonable request.

## Abbreviations

ACR: Albumin/creatinine ratio
AKI: Acute Kidney Injury
BMI: Body Mass Index
CKD: Chronic Kidney Disease
CKDu: Chronic Kidney Disease Of Unknown Cause
DEGREE: Disadvantaged Populations eGFR Epidemiology
eGFR: Estimated glomerular filtration rate
