# Supplementary material for: Prevalence and risk factors for impaired kidney function in the district of Anuradhapura, Sri Lanka: a cross-sectional population-representative survey in those at risk of chronic kidney disease of unknown aetiology
Source: BMC Public Health. 2019 Jun 14;19:763. doi: 10.1186/s12889-019-7117-2 (PMC6570843; doi:10.1186/s12889-019-7117-2)
Supplement: Supplementary file 1 — Questionnaire. Questionnaire used to collate information necessary to development the research. (PDF 195 kb) [file 12889_2019_7117_MOESM1_ESM.pdf]

Cross-sectional Survey to estimate the burden and to understand the aetiology of CKDu in Sri Lanka

Serial Number : ……………………………
GN area : ……………………………
Data collector's Number : ……………………………
Date : …………………………..
GPS mechine number : …………………………..
GPS Coordinates : …………………………..
…………………………..

Name : …………………………………………………………
Address : ………………………………………………….
Telephone Numbers
Land : ……………………………………
Mobile : ………………………………
NIC No : ……………………………………………………

Section 1

Demographic and socio-economic information of the participant

1. Sex *of the respondent* : Female / Male

2. What is your date of birth? : ……………………………… (If unable to remember the exact date of birth, obtain the information from the national identity card.)

3. What is your ethnicity:
   - Sinhala ☐
   - Tamil ☐
   - Muslim ☐
   - Other ……………………………………

4. What is your religion
   - Buddhism ☐
   - Islam ☐
   - Other ………………………………………………….
   - Hindu ☐
   - Roman Catholic / Christian ☐

5. What is the highest level of education you have completed?
   - No formal Schooling ☐
   - 6-11 Grade ☐
   - GCE A/L passed ☐
   - Degree ☐
   - 1-5 Grade ☐
   - GCE O/L passed ☐
   - Certificate/Diploma ☐

   5.1. In total, how many years have you spent at school and in full time study (excluding pre-school)? …… years

6. What is your Marital Status :

Married ☐	Unmarried ☐	Divorced ☐	Widowed ☐

7. What is your current employment status?
   - Full-Time – Public Sector ☐
   - Self-Employed ☐
   - Student ☐
   - Retired ☐
   - Full Time- Private Sector ☐
   - Casual Employment ☐
   - Unpaid Family Work ☐
   - Unemployed ☐

   7.1. If you are working what is your main occupation? ……………………………………………….

   7.2. Considering the nature of your current and previous employments, what of the following describes your employment best (Multiple answers are possible)
   - I have never done an occupation ☐
   - Involves a lot of physical exertion (eg: farming, laborer) ☐
   - Involves working with chemicals (eg: farming, working at a chemical factory) ☐
   - Involves working outdoors in the sun during most part of the day (eg: farming, laborer) ☐
   - Other ☐

7.3. Have you ever migrated to overseas for work? Yes / No

7.3..1. If yes, indicate the country/s and duration/s

Country	Duration

7.4. Have you ever migrated to another part of Sri Lanka for more than one year duration for some reason (work, study) ?

Yes / No

7.4..1. If yes, indicate the area/s and duration

Area	Duration

8. Have ever engaged in the following farming as the main or part time occupation? Yes / No (Go to Q 8.3)

8.1. How long have you been involved in farming? ……………………………. Years

8.2. What are the types of farming you do/did?

**No**	**Type of farming (a)**		**For own consumption only**	**For own consumption & sale**	**Duration of years (b)**
I	Paddy farming	Yes / No	Yes / No	Yes / No	
ii	Subsistence vegetable farming (other than Chena cultivation)	Yes / No	Yes / No	Yes / No	
iii	Chena cultivation	Yes / No	Yes / No	Yes / No	
iv	Other cultivations ……………. …………………………………………..	Yes / No	Yes / No	Yes / No	

8.3. Have you ever done any other occupation? Yes / No (Go to Q 9)

**No**	**Type of occupation**		**For own consumption only**	**For own consumption & sale**	**Duration of years (b)**
I	Fisheries	Yes	Yes / No	Yes / No	
		No			
ii	Other soil & water related industry (Name ……………………..)	Yes			
		No			
iii	Manufacturing industry Specify …………………………	Yes			
		No			

9. What is your average monthly **personal** income? *(if the income is not on monthly basis estimate a monthly amount based on the amount and frequency reported)*

- No income at all ☐
- Rs.5,000-10,000 ☐
- Rs.15,001-20,000 ☐
- Rs.25,001-30,000 ☐
- Less than Rs.5,000 ☐
- Rs.10,001-15,000 ☐
- Rs.20,001-25,000 ☐
- More than Rs.30,000 ☐

10. What is your average monthly **family** income? *(if the income is not on monthly basis estimate a monthly amount based on the amount and frequency reported)*

- No income at all ☐
- Less than Rs.10,000 ☐
- Rs.10,001-20,000 ☐
- Rs.20,001-30,000 ☐
- Rs. 30,001-40,000 ☐
- Rs.40,001-50,000 ☐
- Rs. 50,001-60,000 ☐
- More than Rs.60,000 ☐

Section 2

11. Now I would like to ask you some details of illnesses that you may have. Please mention whether you suffer from any of the following diseases. If you have any, please provide me any diagnosis cards/ clinic records/ clinic books or any other document regarding this. Further, I would like to request your permission to take photographs of the records and they will only be used for the study purpose only.

	**Should be filed by the data collector. Always take a photograph to confirm the record. When you take a photograph always keep the serial number visible.**				**Should be filled after referring to the relevant photograph/s**
	**Disease**	**Have you ever been told that you have this disease? (a)**	**Age at diagnosis (b)**	**(c)**	**Is this confirmed by medical records (diagnosis card/clinic book/ drug sheets) (b)**
I	*Diabetes Mellitus/ DM*	Yes<br>No		Are you taking any drugs for the disease (Yes/No)<br>Took a photograph (Yes/No)	Yes<br>No
ii	*Hypertension/ HT/ HBP*	Yes<br>No		Are you taking any drugs for the disease (Yes/No)<br>*(Take a photograph of the drug list)*<br>Took a photograph (Yes/No)	Yes<br>No<br>*If yes, how many drugs are taking?* ……………………
iii	*Ischaemic Heart Disease/IHD/MI/ CVD*	Yes<br>No		Are you taking any drugs for the disease (Yes/No)<br>Took a photograph (Yes/No)	Yes<br>No
viii	Stroke/ TIA	Yes<br>No		Are you taking any drugs for the disease (Yes/No)<br>Took a photograph (Yes/No)	Yes<br>No
ix	Mental Disorders<br>*Depression*<br>*Anxiety*<br>*Schizophrenia*	Yes<br>No		Are you taking any drugs for the disease (Yes/No)<br>Took a photograph (Yes/No)	Yes<br>No
x	*Tuberculosis/ TB*	Yes<br>No		Are you taking any drugs for the disease (Yes/No)<br>Took a photograph (Yes/No)	Yes<br>No
xi	Cancer/ Carcinoma	Yes<br>No		Are you taking any drugs for the disease (Yes/No)<br>Took a photograph (Yes/No)	Yes<br>No
xii	*Hepatitis B or C)*	Yes<br>No		Are you taking any drugs for the disease (Yes/No)<br>Took a photograph (Yes/No)	Yes<br>No
xiii	HIV/ AIDS	Yes<br>No		Are you taking any drugs for the disease (Yes/No)<br>Took a photograph (Yes/No)	Yes<br>No
xiv	Leptospirosis	Yes<br>No		Are you taking any drugs for the disease (Yes/No)<br>Took a photograph (Yes/No)	Yes<br>No

12. Now I would like to ask you some details of kidney diseases that you may have. Please mention whether you suffer from any of the following diseases. If you have any, please provide me any diagnosis cards/ clinic records/ clinic books or any other document regarding this. Further, I would like to request your permission to take photographs of the records and they will only be used for the study purpose only.

	**Should be filed by the data collector. Always take a photograph to confirm the record. When you take a photograph always keep the serial number visible.**				**Should be filled after referring to the relevant photograph/s**
	**Disease**	**Have you ever been told that you have this disease? (a)**	**Age at diagnosis (b)**	**Take photographs if there are medical records to confirm the diagnosis (c)**	**Is this confirmed by medical records (diagnosis card/clinic book/ drug sheets) (b)**
I	Polycystic kidney	Yes		Took a photograph (Yes/No)	Yes
		No			No
ii	Congenital malformation of kidneys	Yes		Took a photograph (Yes/No)	Yes
		No			No
iii	Obstructive nephropathy	Yes		Took a photograph (Yes/No)	Yes
		No			No
iv	Kidney Stones	Yes		Took a photograph (Yes/No)	Yes
		No			No
v	Glomerulonephritis	Yes		Took a photograph (Yes/No)	Yes
		No			No

12.1. Have you been diagnosed as having Chronic Kidney Disease? Yes / No (go to Q.14)

- Medical record not available Yes / No

12.2. Currently are you registered in a renal clinic Yes / No

- *If "yes" please take a picture of the diagnosis card /clinic book or any other evidence available*

12.3. According to the patient's perception to which of the following category does he belong (Multiple answers are possible)

- Chronic Kidney Disease ☐
- Investigating for Chronic Kidney Disease ☐
- Diabetes Induced CKD ☐
- Hypertension Induced CKD ☐
- Calculi Induced CKD ☐
- CKD of unknown origin ☐
- Other ☐

13. Regarding the treatment you receive for your current kidney disease (Multiple answers are possible)

		Duration of treatment	Are you satisfied with the treatment you receive
Government allopathic hospital	Yes / No		Yes / No
Government aurweda hospital	Yes / No		Yes / No
Traditional medicine	Yes / No		Yes / No
Other (Specify)	Yes / No		Yes / No
Are you currently on dialysis	Yes / No	Years .... months	Yes / No
Didn't do any treatment	Yes / No	If yes, give the reason :	

14. Does anyone of your immediate family members (father/ Mother/ brothers/ sisters) have any of the following diseases? (Multiple answers are possible)

• Chronic Kidney Disease	☐	• Hypertension	☐
• Diabetes	☐	• Ischaemic Heart Disease	☐
• Kidney stones	☐	• Nothing	☐

Section 3

15. Now I would like to ask you some details of the drugs that you may be taking. Please mention whether you are taking any of the drugs mentioned below. If you are using, please provide me any diagnosis cards/ clinic records/ clinic books or any other document regarding this.

<table>
<tr><td></td><td colspan="3">Should be filed by the data collector. Always take a photograph to confirm the record. When you take a photograph always keep the serial number visible.</td><td rowspan="2">Take photographs if there are medical records to confirm
(c)</td><td>Should be filled after referring to the relevant photograph/s</td></tr>
<tr><td></td><td>Drug</td><td>Have you ever been taking the following drugs on a regular basis
(a)</td><td>Duration in years
(b)</td><td>Is this confirmed by medical records (diagnosis card/clinic book/ drug sheets)
(d)</td></tr>
<tr><td rowspan="2">I</td><td rowspan="2">Pain killers like paracetamol</td><td>Yes</td><td rowspan="2"></td><td rowspan="2">Took a photograph (Yes/No)</td><td>Yes</td></tr>
<tr><td>No</td><td>No</td></tr>
<tr><td colspan="6">Regarding the drugs which you are taking daily</td></tr>
<tr><td rowspan="2">ii</td><td rowspan="2">Amitriptyline</td><td rowspan="8">Take a photograph of the currently using drug list.</td><td rowspan="2"></td><td rowspan="2">Took a photograph (Yes/No)</td><td>Yes</td></tr>
<tr><td>No</td></tr>
<tr><td rowspan="2">iii</td><td rowspan="2">Lithium</td><td rowspan="2"></td><td rowspan="2">Took a photograph (Yes/No)</td><td>Yes</td></tr>
<tr><td>No</td></tr>
<tr><td rowspan="2">iv</td><td rowspan="2">Benzodiazepines</td><td rowspan="2"></td><td rowspan="2">Took a photograph (Yes/No)</td><td>Yes</td></tr>
<tr><td>No</td></tr>
<tr><td rowspan="2">v</td><td rowspan="2">Angiotensin-converting enzyme inhibitors</td><td rowspan="2"></td><td rowspan="2">Took a photograph (Yes/No)</td><td>Yes</td></tr>
<tr><td>No</td></tr>
</table>

**State the Aurweda or traditional medicines taken regularly for some time**	
Disease	Duration

16. Now I would like to ask you some details about your diet. Please consider the ***last 7 days*** and state how frequently had the following food items either at home or outside the home. Always the number of days of food consumption is considered (min =0 and max =7)

**No**	**Food item**	**Please state the number of days you consumed the food item during the last week. Circle the answer**							
		0	1	2	3	4	5	6	7
1	Lotus root								
2	Water Lily seed powder								
3	Kohila								
4	Spanich								
5	Cucumber								
6	Olu seeds								
7	Lake fish								
8	Star fruit								
9	Meat (beef, pork or any other red meat)								
10	Chicken								

17. Now I am going to ask you some questions about tobacco use.
    17.1. In the past, did you ever smoke any tobacco products? Yes / No (go to Q.18)
    17.2. Roughly, how many cigarettes have you smoked during your life time?
    - More than 100 (Go to Q 17.3) ☐
    - Less than 100 (Go to Q 18) ☐

    17.3. Do/did you currently / during past 30 days smoke cigarettes? Yes / No (go to Q.18)
    17.4. Do you currently / during past 30 days smoke cigarettes daily or occasionally?
    - Daily ☐
    - Occasionally ☐

18. Now I am going to ask you some questions about smokeless tobacco use [such as chewing tobacco, betel with tobacco, babul, snuff]
    18.1. Do you **currently (during past 30 days)** use any smokeless **tobacco** products? Yes / No (go to Q.18.3)
    18.2. Do you currently **(during past 30 days)** use smokeless tobacco products daily or occasionally?
    - Daily (go to Q.19) ☐
    - Occasionally (go to Q.19) ☐

    18.3. In the past, did you ever use smokeless tobacco products? Yes / No

19. Now I am going to ask you some questions about alcohol use (such as arrack, kasippu, toddy, beer, spirits or wine).
    19.1. Do you currently consume alcohol Yes / No (go to Q.19.3)
    19.2. Do you currently consume alcohol daily Yes (go to Q.20)/ No (go to Q.20)
    19.3. Have you ever consume alcohol Yes / No (go to Q.21)
    19.4. During that period, did you consume alcohol daily Yes / No

20. During the past 12 months, how frequently have you had at least one standard alcoholic drink?
    - Daily ☐
    - 5-6 days per week ☐
    - 3-4 days per week ☐
    - 1-2 days per week ☐
    - 1-3 days per month ☐
    - Less than once a month ☐
    - Not at all ☐
    - Refused ☐

21. The following questions are about your water source.
    21.1. Please state your main water source used for <u>drinking purposes</u>. Further, state how long you have been using each water source. You may have multiple water sources.

No	Source	Duration of use					Rank the water sources according to the most used to least used
		< 5 years	6-10 yrs	11-15 yrs	16-20 yrs	> 20 yrs	
1	Deep Well						
2	Shallow wells						
3	Tube well						
4	Pipe water from water board						
5	Water from reservoirs						
6	Community Based water supply						
7	Farming wells						
8	Canals						
9	Filtered water 9RO filters)						
10	Other						

21.2. Please state your main water source used for <u>cooking purposes</u>. Further, state how long you have been using each water source. You may have multiple water sources.

No	Source	Duration of use					Rank the water sources according to the most used to least used
		< 5 years	6-10 yrs	11-15 yrs	16-20 yrs	> 20 yrs	
1	Deep Well						
2	Shallow wells						
3	Tube well						
4	Pipe water from water board						
5	Water from reservoirs						
6	Community Based water supply						
7	Farming wells						
8	Canals						
9	Filtered water 9RO filters)						
10	Other						

21.3. Usually how much of water do you consume per day? ……………………………. bottles (number of 500ml bottles)

21.4. Curranty are you engaged in farming? Yes / No (go to Q.24)

21.5. If you are a farmer or a person working outdoors during most part of the day, please state your main water source for drinking when you are at work.

- From a well near the paddy field ☐
- Take water from home ☐
- From a "Liyadda" near the field ☐
- Other ☐

22. Have you ever been exposed to chemical fertilizers/weedicides/ pesticides? Yes / No (skip to Q.23)

Please state the type and the duration of exposure of the following chemical fertilizers/weedicides/ pesticides

No	Type	Number of months exposed per yr	Frequency of exposure			Duration of exposure		
			Rarely	Sometimes	Often	< 5 yrs	5-10 yrs	>10 yrs
1	chemical fertilizers							
2	weedicides							
3	pesticides							
4	Other							

23. When you are using chemical fertilizers/weedicides/ pesticides, do you wear personnel protective equipments? Yes / No

23.1. If the answer is “Yes”, how often do you use them? Always / Occasionally

23.2. If the answer is “No”, why don’t you use them? (multiple answers possible)

- No money to buy the equipments ☐
- There is no point using them ☐
- It’s an extra hassle to use them ☐
- Know the importance but lazy to use them ☐
- I don’t know about personnel protective equipments ☐

24. Do you usually work outdoors in the sun? Yes / No (Go to Q.25)

24.1. On a typical working day how many hours do you usually work under the sun? ………………………… Hrs

24.2. How many such days per week do you work under the sun? ……………………………

25. Have you ever been bitten by a snake? Yes / No (Refer to the clinic)

**No**	**Snake**	**Year**	**Needed hospital admission**	**Management at hospitalization (Use the relevant code)**	**Other treatment methods (traditional healers, aurweda, other)**
1	Cobra				
2	Viper				
3	Common krait				
4	Kuna katuwa				
5	Scorpion				
6	Other				

Management at hospitalization

1. Only observed at Primary Care Unit
2. Treated in the ward
3. Treated in the ICU/special unit

Cross-sectional Survey to estimate the burden and to understand the aetiology of CKDu in Sri Lanka

Serial Number : ……………………………
Date : ……………………………

Name : …………………………………………………………
Address : ……………………………………………………
Telephone Numbers
Land : ……………………………………
Mobile : ………………………………
NIC No : ……………………………………………………

Section 4

<table>
<tr><td>Data collectors number</td><td></td><td colspan="3"></td></tr>
<tr><td>Height (m)
…………………………………</td><td></td><td colspan="3">Device ID : ……………………………</td></tr>
<tr><td>Weigjt (kg)
…………………………………</td><td></td><td colspan="3">Device ID: ……………………………</td></tr>
<tr><td rowspan="5">Bioimpedence</td><td rowspan="5"></td><td colspan="3">Device ID: ……………………………</td></tr>
<tr><td colspan="3">Have you eaten yet today? Yes / No</td></tr>
<tr><td colspan="3">Fat percentage of the body : ………………………………… %</td></tr>
<tr><td colspan="3">Water percentage of the body : ………………………………… %</td></tr>
<tr><td colspan="3">Body Mass Index : ………………………………… kg/m^2</td></tr>
<tr><td rowspan="5">Blood Pressure
(mmHg)</td><td rowspan="4"></td><td colspan="3">ළප Device ID : ……………………………</td></tr>
<tr><td>1st reading</td><td>2nd reading</td><td>3rd reading</td></tr>
<tr><td>Higher value</td><td>Higher value</td><td>Higher value</td></tr>
<tr><td>Lower value</td><td>Lower value</td><td>Lower value</td></tr>
<tr><td colspan="3">During the past two weeks, have you been treated for raised blood pressure (medication) prescribed by a doctor/ health worker/?</td><td>Yes / No</td></tr>
<tr><td>Blood glucose level</td><td></td><td colspan="3">______________________________mg /dl</td></tr>
<tr><td>Blood taken for investigations</td><td></td><td colspan="3">Yes / No</td></tr>
<tr><td>Urine taken for investigations</td><td></td><td colspan="3">Yes / No</td></tr>
</table>
