# Supplementary material for: Prevalence and risk factors for impaired kidney function in the district of Anuradhapura, Sri Lanka: a cross-sectional population-representative survey in those at risk of chronic kidney disease of unknown aetiology
Source: BMC Public Health. 2019 Jun 14;19:763. doi: 10.1186/s12889-019-7117-2 (PMC6570843; doi:10.1186/s12889-019-7117-2)
Supplement: Supplementary file 2 — Exclusion criteria. Table detailing the prevalence of the exclusion criteria by sex and study area (DOCX 29 kb) [file 12889_2019_7117_MOESM2_ESM.docx]

**Additional file 2: Prevalence of the exclusion criteria by sex and study area**

| **Presence** | **Area 1**  **(n= 908)** | | **Area 2**  **(n= 1008)** | | **Area 3**  **(n= 926)** | | **Area 4**  **(n= 1000)** | | **Area 5**  **(n= 961)** | | **Total**  **(n= 4803)** | |
| --- | --- | --- | --- | --- | --- | --- | --- | --- | --- | --- | --- | --- |
|  | % | Cl | % | Cl | % | Cl | % | Cl | % | Cl | % | Cl |
| **Presence of possible hypertension (n=1267)**  Blood pressure more than 140/90 at the time of survey (n=785)  Those who were on at least one anti-hypertensive drugs one drug and BP <140/90 **(n=249)**  Patient self-report and not on drugs and BP <140/90 (n=233) | | | | | | | | | | | | |
| Total **(n=4803)** | 26.5 | 23.7 – 29.6 | 26.3 | 23.6 – 29.4 | 26.1 | 23.2 – 29.0 | 30.0 | 27.2 – 32.8 | 22.8 | 20.0 – 25.4 | 26.4 | 25.1 – 27.6 |
| Male **(n=1529)** | 30.7 | 25.6 – 36.1 | 26.2 | 21.6 – 30.9 | 25.2 | 20.7 – 30.0 | 34.3 | 28.7 – 39.4 | 25.2 | 20.2 – 30.3 | 28.3 | 26.1-30.5 |
| Female **(n=3274** | 24.6 | 21.3 – 27.9 | 26.3 | 23.2 – 29.7 | 26.6 | 23.1 – 30.0 | 28.1 | 24.6 – 31.5 | 21.8 | 18.7 – 25.0 | 25.5 | 24.0 – 27.1 |
| **Presence of possible diabetes mellitus (n=470)**  Patients who had a history of diabetes but had normal CBS during the survey (n=226)  Patients who did not have a history of diabetes but had high CBS (>200) during the survey (n=92)  Patients who had a history of diabetes and had high CBS (>200) during the survey (n=152) | | | | | | | | | | | | |
| Total **(n=4803)** | 10.9 | 8.8 – 12.9 | 13.2 | 11.1 – 15.3 | 9.2 | 7.4 – 11.1 | 8.3 | 6.5 – 10.0 | 7.1 | 5.4 – 8.7 | 9.7 | 8.9 – 10.6 |
| Male **(n=1529)** | 9.3 | 5.9 – 12.7 | 12.9 | 9.3 – 16.5 | 8.3 | 5.2 – 11.3 | 8.7 | 5.4 – 11.7 | 6.2 | 3.4 – 9.1 | 9.2 | 7.7 – 10.6 |
| Female **(n=3274** | 11.6 | 9.1 – 14.1 | 13.4 | 10.8 – 16.0 | 9.7 | 7.4 – 12.1 | 8.1 | 6.1 – 10.7 | 7.4 | 5.4 – 9.3 | 10.0 | 9.0 – 11.0 |
| Presence of heavy proteinuria (n=110) | | | | | | | | | | | | |
| Total **(n=4803)** | 1.9 | 1.1 – 2.0 | 2.6 | 1.7 – 3.7 | 2.4 | 1.5 – 3.5 | 1.3 | 0.7 – 2.2 | 3.3 | 2.3 – 4.6 | 2.2 | 1.9 – 2.7 |
| Male **(n=1529)** | 2.4 | 1.1 – 4.9 | 4.1 | 2.4 – 6.8 | 4.1 | 2.4 – 7.0 | 1.6 | 0.7 – 3.9 | 4.5 | 2.6 – 7.6 | 3.4 | 2.6 – 4.4 |
| Female **(n=3274** | 1.6 | 0.8 – 2.9 | 1.7 | 1.0 – 3.1 | 1.4 | 0.7 – 2.7 | 1.1 | 0.5 – 2.2 | 2.8 | 1.8 – 4.3 | 1.7 | 1.3 – 2.2 |
