# Supplementary material for: Prevalence and risk factors for impaired kidney function in the district of Anuradhapura, Sri Lanka: a cross-sectional population-representative survey in those at risk of chronic kidney disease of unknown aetiology
Source: BMC Public Health. 2019 Jun 14;19:763. doi: 10.1186/s12889-019-7117-2 (PMC6570843; doi:10.1186/s12889-019-7117-2)
Supplement: Supplementary file 3 — Age-standardized prevalence rates of CKDu. Table detailing the prevalence of low eGFR in the absence of hypertension, diabetes and proteinuria by sex, age and the study (DOCX 34 kb) [file 12889_2019_7117_MOESM3_ESM.docx]

**Additional file 3: Prevalence of low eGFR in the absence of hypertension, diabetes and proteinuria by sex, age and the study**

|  | **Area 1 (n= 629)** | | | **Area 2 (n= 689)** | | | **Area 3 (n= 650)** | | | **Area 4 (n= 672)** | | | **Area 5 (n= 711)** | | | **Total (n= 3351)** | | |
| --- | --- | --- | --- | --- | --- | --- | --- | --- | --- | --- | --- | --- | --- | --- | --- | --- | --- | --- |
| eGFR mL/min/1.7m^2^ | <30 | 30-60 | 60-90 | <30 | 30-60 | 60-90 | <30 | 30-60 | 60-90 | <30 | 30-60 | 60-90 | <30 | 30-60 | 60-90 | <30 | 30-60 | 60-90 |
|  | n (%) | n (%) | n (%) | n (%) | n (%) | n (%) | n (%) | n (%) | n (%) | n (%) | n  (%) | n (%) | n (%)% | n (%) | n (%) | n (%)% | n (%) | n (%) |
| Age | Male population | | | | | | | | | | | | | | | | | |
| < 25  (n=84) | 0  (0.0) | 0  (0.0) | 0  (0.0) | 0  (0.0) | 0  (0.0) | 0  (0.0) | 0  (0.0) | 0  (0.0) | 0  (0.0) | 0  (0.0) | 0  (0.0) | 0  (0.0) | 0  (0.0) | 0  (0.0) | 3  (23.1) | 0  (0.0) | 0  (0.0) | 3  (3.6) |
| 25-34  (n=158) | 0  (0.0) | 0  (0.0) | 5  (14.7) | 0  (0.0) | 0  (0.0) | 7  (16.3) | 0  (0.0) | 0  (0.0) | 5  (17.9) | 0  (0.0) | 0  (0.0) | 8  (34.8) | 0  (0.0) | 0  (0.0) | 6  (20.0) | 0  (0.0) | 0  (0.0) | 31 (19.6) |
| 35-44  (n=289) | 0  (0.0) | 0  (0.0) | 10 (23.3) | 0  (0.0) | 3  (4.2) | 17 (23.9) | 1  (1.6) | 0  (0.0) | 21 (34.4) | 0  (0.0) | 3  (5.9) | 26 (51.0) | 0  (0.0) | 1  (1.6) | 24 (38.1) | 1  (0.3) | 7  (2.4) | 98 (33.9) |
| 45-54  (n=242) | 1  (3.1) | 1  (3.1) | 20 (62.5) | 1  (1.7) | 4  (6.8) | 27 (45.8) | 6 (10.3) | 5  (8.6) | 22 (37.9) | 2  (4.7) | 3  (7.0) | 33 (76.7) | 0  (0.0) | 5 (10.0) | 24 (48.0) | 10  (4.1) | 18  (7.4) | 126 (52.1) |
| 55-64  (n=162) | 0  (0.0) | 3  (8.6) | 20 (57.1) | 5 (21.7) | 2  (8.7) | 8  (34.8) | 3  (7.3) | 6 (14.6) | 25 (61.0) | 1  (2.8) | 11 (30.6) | 20 (55.6) | 2  (7.4) | 8 (29.6) | 15 (55.6) | 11  (6.8) | 30 (18.5) | 88 (54.3) |
| 65-74  (n=83) | 1  (4.0) | 2  (8.0) | 16 (64.0) | 1  (6.6) | 3  (20.0) | 9  (60.0) | 1  (9.0) | 2 (18.1) | 6  (54.5) | 4  (28.5) | 4  (28.5) | 5  (35.7) | 5  (27.7) | 5  (27.7) | 7  (38.8) | 12 (14.4) | 16 (19.2) | 43 (51.8) |
| => 75  (n=19) | 1  (16.6) | 2  (33.3) | 3  (50.0) | 0  (0.0) | 0  (0.0) | 0  (0.0) | 2  (100.0) | 0  (0.0) | 0  (0.0) | 0  (0.0) | 5  (71.4) | 2  (28.5) | 1  (33.3) | 0  (0.0) | 2  (66.6) | 4  (21.0) | 7  (36.8) | 7  (36.8) |
| Age-standardized (95% CI) * | 0.8 (0.2-1.4) | 2.1 (1.3-2.9) | 29.1 (26.8-31.4) | 3.3 (2.3-4.2) | 4.4 (2.7-6.0) | 24.0 (21.1-26.9) | 3.5 (2.2-4.8) | 4.4 (2.6-6.2) | 27.8 (24.6-31.0) | 3.2 (1.1-5.2) | 7.9 (5.9-10.0) | 39.0 (36.7-41.3) | 2.9 (1.1-4.7) | 7.4 (5.6-9.2) | 34.3 (32.2-36.4) | 2.6 (2.0-3.2) | 5.2 (4.5-5.9) | 30.2 (29.1-31.4) |
| Age | Female population | | | | | | | | | | | | | | | | | |
| < 25  (n=232) | 0  (0.0) | 0  (0.0) | 1  (2.4) | 0  (0.0 | 1  (1.9) | 3  (5.8) | 0  (0.0) | 0  (0.0) | 0  (0.0) | 0  (0.0) | 0  (0.0) | 1  (2.0) | 0  (0.0) | 0  (0.0) | 0  (0.0) | 0  (0.0) | 1  (0.4) | 5  (2.2) |
| 25-34  (n=580) | 0  (0.0) | 0  (0.0) | 11 (10.7) | 0  (0.0 | 0  (0.0 | 12 (10.6) | 0  (0.0) | 1  (0.9) | 11  (9.4) | 0  (0.0) | 0  (0.0) | 34  (25.0) | 0  (0.0) | 0  (0.0) | 16 (14.4) | 0  (0.0) | 1  (0.2) | 84 (14.5) |
| 35-44  (n=708) | 0  (0.0) | 0  (0.0) | 40 (30.1) | 0  (0.0 | 5 (3.8) | 33 (25.2) | 0  (0.0) | 0  (0.0) | 35 (27.3) | 0  (0.0) | 3  (2.1) | 80 (56.3) | 0  (0.0) | 3  (1.7) | 63 (36.2) | 0  (0.0) | 11  (1.6) | 251 (35.5) |
| 45-54  (n=385) | 0  (0.0 | 2  (2.9) | 43 (61.4) | 1  (1.3) | 3 (3.9) | 41 (53.2) | 0  (0.0) | 2  (2.8) | 27  (38) | 0  (0.0) | 3  (3.6) | 53 (63.1) | 0  (0.0) | 7  (8.4) | 48 (57.8) | 1  (0.3) | 17  (4.4) | 212 (55.1) |
| 55-64  (n=273) | 1  (1.7) | 2  (3.4) | 38 (64.4) | 0  (0.0 | 2 (3.6) | 35 (63.6) | 0  (0.0) | 3  (6.7) | 36  (80.0) | 0  (0.0) | 6 (13.0) | 33 (71.7) | 1  (1.5) | 5  (7.4) | 50 (73.5) | 2  (0.7) | 18 (6.6) | 192 (70.3) |
| 65-74  (n=113) | 3 (11.5) | 1  (3.8) | 17 (65.3) | 1  (4.7) | 4 (19.0) | 13 (61.9) | 0  (0.0) | 2  (8.6) | 20 (86.9) | 0  (0.0) | 8 (42.1) | 9  (47.3) | 1  (4.1) | 3 (12.5) | 19 (79.1) | 5  (4.4) | 18 (15.9) | 78 (69.0) |
| => 75  (n=23) | 0  (0.0) | 3 (50.0) | 3  (50.0) | 1 (25.0) | 2 (50.0) | 1  (25.0) | 0  (0.0) | 1 (50.0) | 1 (50.0) | 1 (16.6) | 3 (50.0) | 2  (33.3) | 1 (20.0) | 0  (0.0) | 3  (60.0) | 3  (13.0) | 9 (39.1) | 10 (43.4) |
| Age-standardized (95% CI) * | 1.0 (0.1-2.0) | 1.1 (0.6-1.7) | 31.2 (28.9-33.5) | 0.6  (-0.1-1.2) | 3.6 (2.3-5.0) | 29.4 (26.9-31.9) | 0  (0.0) | 2.1 (1.2-2.9) | 29.5 (26.6-32.20 | 0  (0.0) | 5.6 (3.4-7.7) | 39.1 (36.8-41.4) | 0.5 (-0.1-1.1) | 3.5 (2.4-4.5) | 34.1 (31.5-36.8) | 0.5 (0.2-0.7) | 3.1 (2.6-3.6) | 33.0 (31.834.1) |

*Age-standardized (95% CI) - excluding the age category >=75 years
