# Supplementary material for: Prevalence and risk factors for impaired kidney function in the district of Anuradhapura, Sri Lanka: a cross-sectional population-representative survey in those at risk of chronic kidney disease of unknown aetiology
Source: BMC Public Health. 2019 Jun 14;19:763. doi: 10.1186/s12889-019-7117-2 (PMC6570843; doi:10.1186/s12889-019-7117-2)
Supplement: Supplementary file 4 — CKDu prevalence according to occupation and lifestyle factors. Table detailing the prevalence of eGFR< 60 according to occupation and lifestyle factors in the absence of hypertension, diabetes and proteinuria by sex (DOCX 28 kb) [file 12889_2019_7117_MOESM4_ESM.docx]

**Additional file 4: Prevalence of eGFR<60 according to occupation and lifestyle factors in the absence of hypertension, diabetes and proteinuria by sex**

| **Prevalance** | **Male** | | | **Female** | | |
| --- | --- | --- | --- | --- | --- | --- |
|  | **n** | **%** | **CI** | **n** | **%** | **CI** |
| **Occupation** |  |  |  |  |  |  |
| Ever occupied in any farming and duration |  |  |  |  |  |  |
| No farming | 179 | 2.2 | 0.04 – 4.4 | 829 | 1.5 | 0.07 – 2.4 |
| Part time farming for < 10 yrs | 145 | 2.7 | 0.06 – 5.4 | 157 | 1.9 | 0.0 – 4.0 |
| Part time farming for ≥ 10 yrs | 296 | 12.5 | 8.7 – 16.3 | 253 | 11.0 | 7.1 – 14.9 |
| Full time farming for < 10 yrs | 57 | 0.0 | 0.0 – 0.0 | 291 | 0.3 | 0.0 – 1.0 |
| Full time farming for ≥ 10 yrs | 360 | 19.7 | 15.6 – 23.8 | 784 | 5.2 | 3.6 – 6.7 |
| Used fertilizers/ Weedicides/ Pesticides | 739 | 12.7 | 10.3 – 15.1 | 699 | 3.2 | 1.9 – 4.6 |
|  |  |  |  |  |  |  |
|  |  |  |  |  |  |  |
| **Life style factors** |  |  |  |  |  |  |
| Ever smoked | 523 | 6.2 | 4.1 – 8.3 | 16 | 25.0 | 1.1 – 48.8 |
| Alcohol ever used | 744 | 12.1 | 9.7 – 14.4 | 43 | 11.6 | 1.6 – 21.6 |
| Deep-wells as the drinking water source | 793 | 11.8 | 9.5 – 14.1 | 1721 | 3.9 | 3.0 – 4.8 |
| Shallow wells as the drinking water source | 84 | 9.5 | 3.1 – 15.9 | 169 | 2.9 | 0.3 – 5.5 |
| Tube well as the drinking water source | 104 | 11.5 | 5.2 – 17.7 | 239 | 4.1 | 1.6 – 6.7 |
| Consumed less than 3 liters of water per day | 407 | 10.5 | 7.5 – 13.5 | 1524 | 3.9 | 2.9 – 4.9 |
| Work outside exposed to the sun less than 20 hours per week | 579 | 11.0 | 8.4 – 13.6 | 616 | 4.0 | 2.4 – 5.6 |
| Bitten by a snake (any snake) | 82 | 17.0 | 8.7 – 25.4 | 92 | 6.5 | 1.4 – 11.6 |
