# Supplementary material for: Prevalence and risk factors for impaired kidney function in the district of Anuradhapura, Sri Lanka: a cross-sectional population-representative survey in those at risk of chronic kidney disease of unknown aetiology
Source: BMC Public Health. 2019 Jun 14;19:763. doi: 10.1186/s12889-019-7117-2 (PMC6570843; doi:10.1186/s12889-019-7117-2)
Supplement: Supplementary file 5 — Sensitivity analysis. Table detailing the results of the sensitivity analysis on the association of eGFR (in the absence of diabetes, or hypertension or heavy protienuria) and ever occupied in of farming (DOCX 35 kb) [file 12889_2019_7117_MOESM5_ESM.docx]

**Additional file 5: Results of the sensitivity analysis on the association of eGFR (in the absence of diabetes, or hypertension or heavy protienuria) and ever occupied in of farming**

| Variables | All | | | | | | | | | Male | | | | | | | | | | | | Female | | | | | | | | | | |
| --- | --- | --- | --- | --- | --- | --- | --- | --- | --- | --- | --- | --- | --- | --- | --- | --- | --- | --- | --- | --- | --- | --- | --- | --- | --- | --- | --- | --- | --- | --- | --- | --- |
|  |  |  | | 95% Confidence Interval for B | | | | |  | | | |  | | | 95% Confidence Interval for B | | | | |  | | | |  | | | 95% Confidence Interval for B | | | | |
|  | β | Sig. | | Lower | | Upper | | β | | | | Sig. | | | Lower | | | Upper | | β | | | | Sig. | | | Lower | | | Upper | |  |
| (Constant) | 103.79 | 0.00 | | 102.43 | | 105.15 | | 106.91 | | | | 0.00 | | | 103.25 | | | 110.57 | | 103.08 | | | | 0.00 | | | 101.71 | | | 104.45 | |  |
| **Ever occupied in farming** | **-19.08** | **0.00** | | **-20.65** | | **-17.50** | | -24.73 | | | | 0.00 | | | -28.70 | | | -20.76 | | -16.92 | | | | 0.00 | | | -18.58 | | | -15.27 | |  |
| (Constant) | 141.33 | 0.00 | | 139.47 | | 143.18 | | 147.64 | | | | 0.00 | | | 143.41 | | | 151.87 | | 138.36 | | | | 0.00 | | | 136.37 | | | 140.34 | |  |
| **Ever occupied in farming** | **-3.71** | **0.00** | | **-5.13** | | **-2.29** | | -5.95 | | | | 0.00 | | | -9.47 | | | -2.42 | | -3.40 | | | | 0.00 | | | -4.87 | | | -1.94 | |  |
| Sex | -1.26 | 0.04 | | -2.48 | | -0.05 | |  | | | |  | | |  | | |  | |  | | | |  | | |  | | |  | |  |
| Age | -1.07 | 0.00 | | -1.12 | | -1.03 | | -1.19 | | | | 0.00 | | | -1.28 | | | -1.11 | | -1.01 | | | | 0.00 | | | -1.06 | | | -0.96 | |  |
| (Constant) | 165.39 | 0.00 | | 155.24 | | 175.54 | | 201.40 | | | | 0.00 | | | 182.25 | | | 220.55 | | 144.05 | | | | 0.00 | | | 131.68 | | | 156.43 | |  |
| **Ever occupied in farming** | -2.86 | 0.00 | | -4.28 | | -1.44 | | -3.91 | | | | 0.03 | | | -7.39 | | | -0.43 | | -2.86 | | | | 0.00 | | | -4.33 | | | -1.39 | |  |
| Sex | 7.11 | 0.00 | | 5.02 | | 9.20 | |  | | | |  | | |  | | |  | |  | | | |  | | |  | | |  | |  |
| Age | -1.03 | 0.00 | | -1.08 | | -0.99 | | -1.09 | | | | 0.00 | | | -1.18 | | | -1.00 | | -1.00 | | | | 0.00 | | | -1.06 | | | -0.95 | |  |
| Number of years of education | 0.12 | 0.16 | | -0.04 | | 0.28 | | 0.38 | | | | 0.02 | | | 0.05 | | | 0.72 | | 0.00 | | | | 0.97 | | | -0.17 | | | 0.18 | |  |
| Ever smoker | -5.29 | 0.00 | | -7.51 | | -3.06 | | -3.09 | | | | 0.02 | | | -5.79 | | | -0.39 | | -13.48 | | | | 0.00 | | | -21.32 | | | -5.64 | |  |
| Alcohol ever use | -3.54 | 0.00 | | -5.86 | | -1.23 | | -3.21 | | | | 0.03 | | | -6.15 | | | -0.26 | | -4.29 | | | | 0.12 | | | -9.66 | | | 1.09 | |  |
| Body water % | -0.44 | 0.00 | | -0.60 | | -0.29 | | -1.00 | | | | 0.00 | | | -1.28 | | | -0.72 | | -0.04 | | | | 0.71 | | | -0.23 | | | 0.16 | |  |
| BMI | -0.22 | 0.00 | | -0.36 | | -0.07 | | -0.15 | | | | 0.29 | | | -0.42 | | | 0.13 | | -0.14 | | | | 0.09 | | | -0.31 | | | 0.02 | |  |
| Histiry of CKD among parents or siblings | -3.71 | 0.00 | | -4.89 | | -2.53 | | -4.78 | | | | 0.00 | | | -7.19 | | | -2.37 | | -3.40 | | | | 0.00 | | | -4.70 | | | -2.09 | |  |
| (Constant) | 166.42 | 0.00 | | 156.25 | | 176.60 | | 202.15 | | | | 0.00 | | | 182.94 | | | 221.35 | | 145.18 | | | | 0.00 | | | 132.79 | | | 157.58 | |  |
| **Ever occupied in farming** | -2.81 | 0.00 | | -4.23 | | -1.40 | | -3.84 | | | | 0.03 | | | -7.32 | | | -0.36 | | -2.83 | | | | 0.00 | | | -4.30 | | | -1.36 | |  |
| Sex | 7.17 | 0.00 | | 5.08 | | 9.26 | |  | | | |  | | |  | | |  | |  | | | |  | | |  | | |  | |  |
| Age | -1.03 | 0.00 | | -1.07 | | -0.98 | | -1.09 | | | | 0.00 | | | -1.18 | | | -0.99 | | -1.00 | | | | 0.00 | | | -1.05 | | | -0.94 | |  |
| Number of years of education | 0.13 | 0.13 | | -0.04 | | 0.29 | | 0.39 | | | | 0.02 | | | 0.05 | | | 0.72 | | 0.01 | | | | 0.89 | | | -0.16 | | | 0.19 | |  |
| Ever smoker | -5.25 | 0.00 | | -7.47 | | -3.03 | | -3.08 | | | | 0.03 | | | -5.78 | | | -0.38 | | -13.23 | | | | 0.00 | | | -21.06 | | | -5.39 | |  |
| Alcohol ever use | -3.62 | 0.00 | | -5.94 | | -1.31 | | -3.19 | | | | 0.03 | | | -6.13 | | | -0.24 | | -4.92 | | | | 0.07 | | | -10.30 | | | 0.47 | |  |
| Body water % | -0.45 | 0.00 | | -0.61 | | -0.29 | | -1.00 | | | | 0.00 | | | -1.28 | | | -0.72 | | -0.04 | | | | 0.67 | | | -0.24 | | | 0.15 | |  |
| BMI | -0.21 | 0.00 | | -0.36 | | -0.07 | | -0.14 | | | | 0.31 | | | -0.42 | | | 0.13 | | -0.14 | | | | 0.11 | | | -0.31 | | | 0.03 | |  |
| Histiry of CKD among parents or siblings | -3.65 | 0.00 | | -4.83 | | -2.47 | | -4.81 | | | | 0.00 | | | -7.22 | | | -2.40 | | -3.28 | | | | 0.00 | | | -4.59 | | | -1.97 | |  |
| Deep wells as the drinking water source | -1.72 | 0.01 | | -3.01 | | -0.43 | | -1.40 | | | | 0.30 | | | -4.05 | | | 1.25 | | -1.89 | | | | 0.01 | | | -3.31 | | | -0.46 | |  |
| (Constant) | 166.71 | 0.00 | | 156.54 | | 176.87 | | 202.25 | | | | 0.00 | | | 183.04 | | | 221.45 | | 145.34 | | | | 0.00 | | | 132.95 | | | 157.74 | |  |
| **History of Farming** | -2.93 | 0.00 | | -4.42 | | -1.45 | | -4.25 | | | | 0.02 | | | -7.88 | | | -0.62 | | -2.92 | | | | 0.00 | | | -4.47 | | | -1.38 | |  |
| Sex | 7.39 | 0.00 | | 5.29 | | 9.49 | |  | | | |  | | |  | | |  | |  | | | |  | | |  | | |  | |  |
| Age | -1.03 | 0.00 | | -1.07 | | -0.98 | | -1.08 | | | | 0.00 | | | -1.17 | | | -0.99 | | -1.00 | | | | 0.00 | | | -1.05 | | | -0.94 | |  |
| Number of years of education | 0.13 | 0.10 | | -0.03 | | 0.30 | | 0.42 | | | | 0.01 | | | 0.09 | | | 0.76 | | 0.01 | | | | 0.92 | | | -0.17 | | | 0.19 | |  |
| Ever smoker | -5.17 | 0.00 | | -7.39 | | -2.94 | | -2.97 | | | | 0.03 | | | -5.66 | | | -0.27 | | -13.24 | | | | 0.00 | | | -21.09 | | | -5.39 | |  |
| Alcohol ever use | -3.54 | 0.00 | | -5.86 | | -1.22 | | -3.11 | | | | 0.04 | | | -6.06 | | | -0.15 | | -4.84 | | | | 0.08 | | | -10.22 | | | 0.55 | |  |
| Body water % | -0.46 | 0.00 | | -0.61 | | -0.30 | | -1.01 | | | | 0.00 | | | -1.29 | | | -0.73 | | -0.05 | | | | 0.65 | | | -0.24 | | | 0.15 | |  |
| BMI | -0.20 | 0.01 | | -0.34 | | -0.05 | | -0.11 | | | | 0.45 | | | -0.38 | | | 0.17 | | -0.13 | | | | 0.13 | | | -0.30 | | | 0.04 | |  |
| Histiry of CKD among parents or siblings | -3.63 | 0.00 | | -4.81 | | -2.45 | | -4.74 | | | | 0.00 | | | -7.16 | | | -2.33 | | -3.27 | | | | 0.00 | | | -4.57 | | | -1.96 | |  |
| Deep wells as the drinking water source | -1.57 | 0.02 | | -2.86 | | -0.28 | | -0.91 | | | | 0.50 | | | -3.57 | | | 1.75 | | -1.84 | | | | 0.01 | | | -3.27 | | | -0.41 | |  |
| Snake bite (any snake) | -2.55 | 0.01 | | -4.57 | | -0.53 | | -2.54 | | | | 0.17 | | | -6.13 | | | 1.06 | | -2.61 | | | | 0.03 | | | -5.03 | | | -0.19 | |  |
| work outside exposed to the sun | 1.20 | 0.07 | | -0.08 | | 2.47 | | 2.63 | | | | 0.03 | | | 0.26 | | | 5.01 | | 0.60 | | | | 0.43 | | | -0.89 | | | 2.09 | |  |
| Aount of water consumed | -1.38 | 0.02 | | -2.55 | | -0.22 | | -2.74 | | | | 0.02 | | | -5.12 | | | -0.37 | | -0.55 | | | | 0.41 | | | -1.84 | | | 0.74 | |  |
| Use of fertilizers/ Weedicides/ Pesticides | -0.66 | 0.52 | | -2.67 | | 1.34 | | -0.78 | | | | 0.61 | | | -3.83 | | | 2.26 | | 0.35 | | | | 0.81 | | | -2.46 | | | 3.16 | |  |
| a. Dependent Variable: eGFR (CKD-EPI) (in the absence of diabetes, or hypertension or heavy protienuria) | | |  | |  | |  | | | |  | | |  | | |  | |  | | | |  | | |  | | |  | |  | |
